# Dynamic Diagnosis of Familial Prion Diseases Supports the β2-α2 Loop as a Universal Interference Target

**DOI:** 10.1371/journal.pone.0019093

**Published:** 2011-04-28

**Authors:** Massimiliano Meli, Maria Gasset, Giorgio Colombo

**Affiliations:** 1 Department of Computational Biology, Istituto di Chimica del Riconoscimento Molecolare, Consiglio Nazionale delle Ricerche, Milano, Italy; 2 Department of Biological Physical Chemistry, Instituto Química-Física “Rocasolano”, Consejo Superior de Investigaciones Científicas, Madrid, Spain; National Institute for Medical Research, Medical Research Council, London, United Kingdom

## Abstract

**Background:**

Mutations in the cellular prion protein associated to familial prion disorders severely increase the likelihood of its misfolding into pathogenic conformers. Despite their postulation as incompatible elements with the native fold, these mutations rarely modify the native state structure. However they variably have impact on the thermodynamic stability and metabolism of PrP^C^ and on the properties of PrP^Sc^ aggregates. To investigate whether the pathogenic mutations affect the dynamic properties of the HuPrP(125-229) α-fold and find possible common patterns of effects that could help in prophylaxis we performed a dynamic diagnosis of ten point substitutions.

**Methodology/Principal Findings:**

Using all-atom molecular dynamics simulations and novel analytical tools we have explored the effect of D178N, V180I, T183A, T188K, E196K, F198S, E200K, R208H, V210I and E211Q mutations on the dynamics of HuPrP(125-228) α-fold. We have found that while preserving the native state, all mutations produce dynamic changes which perturb the coordination of the α2-α3 hairpin to the rest of the molecule and cause the reorganization of the patches for intermolecular recognition, as the disappearance of those for conversion inhibitors and the emergence of an interaction site at the β2-α2 loop region.

**Conclusions/Significance:**

Our results suggest that pathogenic mutations share a common pattern of dynamical alterations that converge to the conversion of the β2-α2 loop into an interacting region that can be used as target for interference treatments in genetic diseases.

## Introduction

Prion diseases are fatal neurodegenerations of mammals featured by the apparition of misfolded and aggregated forms of the cellular prion protein (PrP^C^) [1],[2]. These forms are collectively referred to as disease-related prion protein (PrP^Sc^) and display self-propagative properties [1],[2]. PrP^Sc^ is formed from PrP^C^ in a posttranslational multistep refolding process, in which the native structure of PrP^C^ partially unfolds to expose regions that direct the assembly of the yet structurally unresolved β-sheet rich and protease resistant PrP^Sc^ molecular aggregates [3]–[9]. Once formed, PrP^Sc^ self-perpetuates by propagating as a template its fold to PrP^C^ [1],[2]. In human familial diseases, mutations in the open-reading frame of *PRNP* produce PrP chains with an increased likelihood for PrP^Sc^ formation [1],[2]. In these cases the late-onset of the disease and the identification of adequate pharmacological targets may allow intervention strategies for asymptomatic carriers directed to retard if not abrogate the pathogenic conversion.

NMR studies have shown that PrP^C^ native state is featured by an N-terminal flexible tail and a C-terminal globular domain (residues 125–228, human sequence numbering), with a fold containing three α-helices and an anti-parallel β-sheet [10]–[13]. These elements are arranged into two halves, β1-α1-β2 and α2-α3, that are packed against each other defining the hydrophobic core [10]–[13]. Of them, the α2-α3 region behaves as an independent folding unit, that displays unique aggregation properties and forms the parallel in-registered β-sheet backbone of the recombinant PrP fibrils [4],[14]–[17]. Despite initial considerations, most pathogenic mutations rarely alter the native structure of PrP^C^, but perturb its stability, regulate its metabolism, modulate the oligomerization pathways and determine the features of the PrP^Sc^ assemblies [2],[4],[14],[18]–[27]. Moreover, molecular dynamics (MD) studies have reported that some mutations cause local conformational effects (as on the β2-α2 and α2-α3 regions) and affect the packing and flexibility properties of the native state [19],[28]–[31]. On the other hand, studies on the effects of oxidative modifications and of the side-chain polarity (e.g. with M-to-S mutations) have shown that perturbations of the native state can transmit long-range and, disturbing the network of stabilizing interactions, facilitate the population of alternative states, some of which may cause the routing into the productive pathogenic conversion [31]–[33]. Interestingly, the long-range propagation of perturbations has also been observed for some mutant PrP chains [19],[33]. Theoretical approaches then suggest that mutations provoke dynamic alterations of the native state and that, by modifying the conformational preferences, may also perturb its molecular recognition properties. Changes in the exposition of binding sites for inhibitors and of segments engaged in aggregation reactions could be crucial for the permissibility of the pathogenic structural transitions. Also, the identification of these regions may provide useful targets for pharmacological interference.

To construct a global view on the role of pathological point mutations on PrP conversion, in this study we have explored the effect of ten mutations occurring at the α2-α3 region (namely D178N, V180I, T183A, T188K, E196K, F198S, E200K, R208H, V210I and E211Q) on the conformational dynamics of the native state and of its consequences on the definition of interaction patches. We have found that while point substitutions adapt quite well to the native structure, they have diverse effects on its dynamic properties. The analysis of these dynamic signatures lead to the identification of the β2-α2 loop as a common possible site for interaction, onto which drug design can be targeted with the goal of interfering with the aggregation process in mutant carriers.

## Results

**Figure 1:**
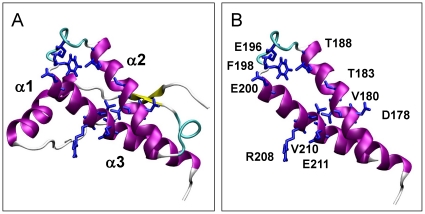
3D structure of the HuPrP(125–228) α-fold used as template for the generation of the different mutants and sulfoxidized variants. (**A**) 3D structure of the HuPrP(125–228) α-fold corresponding to the Pdb entry 1QLZ used as a starting point in the simulations [18] and depicting the different helical structures. (**B**) Side chains replaced for the generation of the different mutants used in this study.

To facilitate the identification of common patterns of behavior and to compare with previous reports, we selected ten point substitutions linked to familial prion diseases and a PrP sequence having methionine at position 129 [**Figure 1** **,** **Table 1**]. These mutations are located in the α2-α3 region of the C-terminal globular domain and include substitutions of residues with both charged and hydrophobic side chains. Of them six are associated with Creutzfeldt-Jakob disease (CJD) [V180I, E196K, E200K, R208H, V210I and E211Q], one with Familial Fatal Insomnia (FFI) [D178N], two with dementia [T183A and T188K] and one with Gerstmann-Straüssler-Scheinker syndrome (GSS) [F198S] [**Figure 1** **,** **Table 1**]. For comparison we included the data from the wild type (wt) and of the M206 and M213 sulfoxidized variants [32]. **Table 1** summarizes the features of these protein chains and the parameters used for the simulations. The obtained trajectories were analyzed in terms of changes on the native state flexibility, the internal dynamics and coordination properties of the fold, the solvent exposure of α3 methionines (M205, M206 and M213) and the energetics of the protein surface in relation to the definition of interaction patches linked to aggregation/self-recognition processes.

**Table 1:** Parameters of the HuPrP(125–228) chains used for the simulation.

HuPrP (125–228)	Charge	Time (ns)	Water molecules	Disease
wt	-3	80.7	7051	
MO206	-3	161.6	6432	
MO213	-3	169.6	7052	
MO206,MO213	-3	160	7051	
D178N	-1	200	7050	FFI^a^
V180I	-3	200	7051	CJD
T183A	-3	200	7050	dementia
T188K	-2	200	7047	dementia
E196K	-1	200	7050	CJD
F198S	-3	190	7057	GSS
E200K	-1	180	7045	CJD
R208H	-4	200	7056	CJD
V210I	-3	200	7051	CJD
E211Q	-2	200	7053	CJD

Mutants and covalent variants were generated by replacement of side chains as indicated in the method section. The disease phenotype generated by the mutation in the *PRNP* gene is also indicated [1],[2].

a Since the template chains has a methionine residue at 129, the phenotype of D178N mutation corresponds to FFI [1],[2].

### Most mutations behave as flexibility amplifiers

**Figure 2:**
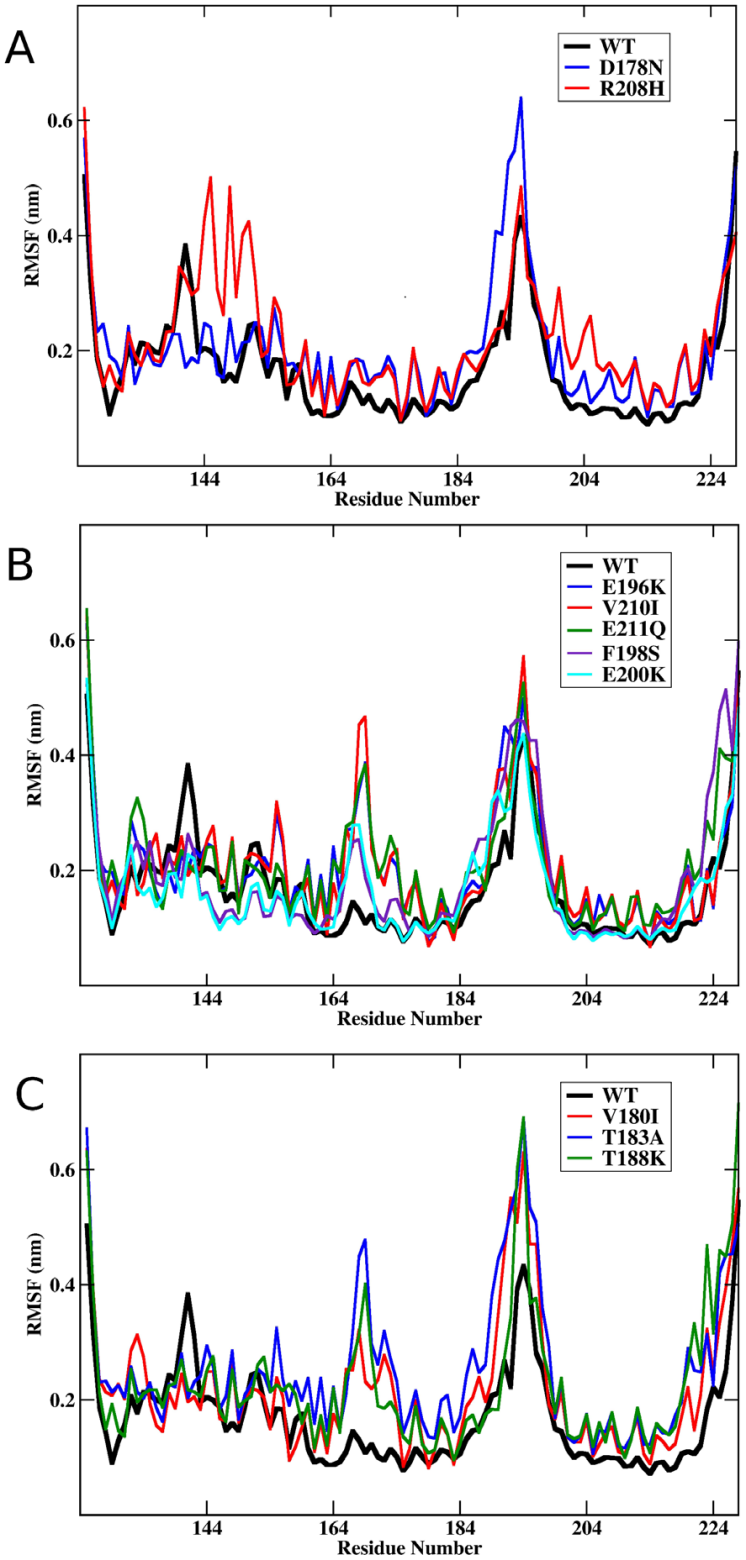
Effect of mutations on the residue based average flexibility. Variation of the Root Mean Square Fluctuations (RMSF) as a function of the residue number for the different substituted protein forms: (**A**) D178N (blue), R208H (red); (**B**) E196K (blue), F198S (purple), E200K (cyan), V210I (red), E211Q (green); and (**C**) V180I (red), T183A (blue), T188K (green). For easiest comparison, the curve corresponding to the wt chain has been included in each panel as a black thick trace.

The calculation of the root mean square fluctuation (RMSF) of each residue around its average position in the respective MD trajectory was used to analyze changes in the protein flexibility. Consistently with previous data [32], the variation in flexibility of the wt native state involves mainly two regions, residues 165–175 and residues 185–200, comprising the β2-α2 loop and the α2-α3 structural regions, respectively. Mutants D178N and R208H show a general wt-like behavior, with only a slight increase between residues 134–156 [**Figure 2A**]. As reported for sulfoxidized variants at positions M206, M213 (MO206, MO213 and MO206–213) [32], the E196K, F198S, E200K, V210I and E211Q mutations determine an enhancement of flexibility that is restricted to the region 165–175 [**Figure 2B**]. Interestingly, the RMSF pattern of both wt and E200K chains obtained here with GROMACS reproduces the flexibility pattern generated with AMBER [29]. Mutants V180I, T183A and T188K show a definite increase of flexibility in both the 165–175 and the 185–200 regions [**Figure 2C**]. In addition to these changes, an increase in the flexibility around residues 220–228 forming part of the C-terminus of α3 is also noticed in the V180I, T183A, T188K, F198S and E211Q mutants. Then, these data supports that in general mutations function as native state flexibility amplifiers and subsequently they can facilitate the access to alternate conformational states.

### Mutations affect the coordination of internal protein dynamics

**Figure 3:**
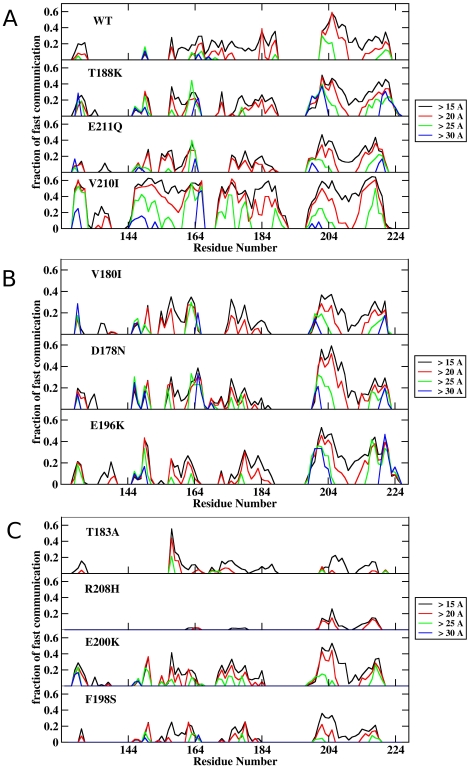
Effect of methionine oxidation on the communication efficiency of all residues at increasing distances. Each bin refers to a residue and shows the fraction of residues of the whole protein that are highly prone to communicate with it (CP<0.025). In each histogram only communications at distances greater than a given threshold: (<15 Å, black lines; >20 Å, red lines; >25 Å, green lines; and >30 Å, blue lines) are considered. From top to bottom: (**A**) wt, T188K, V210I, E211Q; (**B**) D178N, V180I, E196K; and (**C**) T183A, F198S, E200K, R208H.

Since chemical changes such as those arising from mutations can alter the dynamical states and these alterations, if significant, must be distinctly sensed by the residues in different regions of the protein [32],[33], we next analyzed the differential effects of the mutations on the internal dynamics of the protein [**Figure 3**]. For this purpose, we calculated the communication propensity (CP) histograms of the different mutants of the protein [32]. It must be stressed that in these histograms the number of residues that communicate efficiently (y-axis) with a given target residue (x-axis) represents a reverberation of the degree of internal coordination of the residue-residue pairs in a protein structure and consequently it reports on the diffusion of collective dynamic effects due to specific perturbations. In other words, these calculations inform on the capacity of a mutation to transmit a signal and on the identity of the regions sensing it. Furthermore, differences between the mutant and the wt profile may also highlight the capacity of the mutant chain to sampling alternative states leading to the perturbation of the native conformations.

Indeed all mutations impact on the internal coordination of the HuPrP(125–228) α-fold and the type and magnitude of the effect depend on the intrinsic features of the mutation, as its identity and its positioning along the sequence [**Figure 3**]. Interestingly, the dynamic effects of the mutations are not limited only to the sequence stretch in which they are located, but are transmitted long-range through the whole structure. As reported previously and displayed here for an easier visualization of changes, in the native state of wt the segment comprising β2, α2 and their connecting loop is active in communications with α3 in a range of 15 Å. At increasing distances, the communications are progressively reduced and no residue pairs are found to communicate at separating distances larger than 30 Å. Mutations T183A and R208H, which have opposite effects as flexibility amplifiers (largest and none, respectively), almost completely abolish the internal coordination of the protein. This effect was previously reported for an artificial M206S-M213S mutant constructed to mimic the polarity increase caused by sulfoxidation and that is partially unstructured and prone to aggregate in solution [33]. At the opposite side of the spectrum of effects, the T188K mutation determines a large increase in the coordinated motions of residue pairs. However, for this mutant the motions of the 164–174 region appears decoupled from those of the rest of the protein.

Taking all together these data show that mutants share as common features distinct from the wt the loss of coordination involving residues in the 164–174, 185–205 and 218–224 regions, comprising the β2-α2 and α2-α3 loops and the C-terminal part of α3, respectively [**Figure 3**]. Importantly, the β2-α2 loop, links the two halves of the native fold and its sequence behave as a fibrillation motif [8],[9]. Moreover, the other regions affected are contained in the α2-α3 helical hairpin, that behaving as an autonomous folding unit and participating in the fibril backbone of recombinant PrP, when isolated reproduces both the structural transition and aggregation properties of the full length chain [14]–[17]. Then, these data suggest that the lack of coordination of these stretches with the rest of the protein could provide the conformational freedom and flexibility necessary for the subdomain detaching and to ease barrier crossing to states alternative from the native conformation with increased aggregation propensity.

### Mutations increase the solvent exposure of α3 methionines facilitating their oxidation

Previous work has shown that the methionine residues forming part of α3 encrypt a redox code for the α-fold conformational stability, whose role in prion biogenesis is still unclear [32]–[36]. Oxidation of these residues, which among the different methionines in the chain are those less accessible to the solvent, has been found in aggregated PrP forms, albeit without any clear quantitative determination of their amounts and leaving issues open regarding their biological relevance [34]–[36]. Both molecular dynamics and biophysical approaches have shown that the side chain polarity enhancement produced by oxidation impede the native α-fold and provide the structural flexibility required for the acquisition of aggregation properties [31]–[34],[36]. Then, the probability of the α3 methionines to undergo oxidation, which relates to their exposure to oxidants or in general to solvent, can be taken as a index of the acquisition of pro-aggregating features.

**Figure 4:**
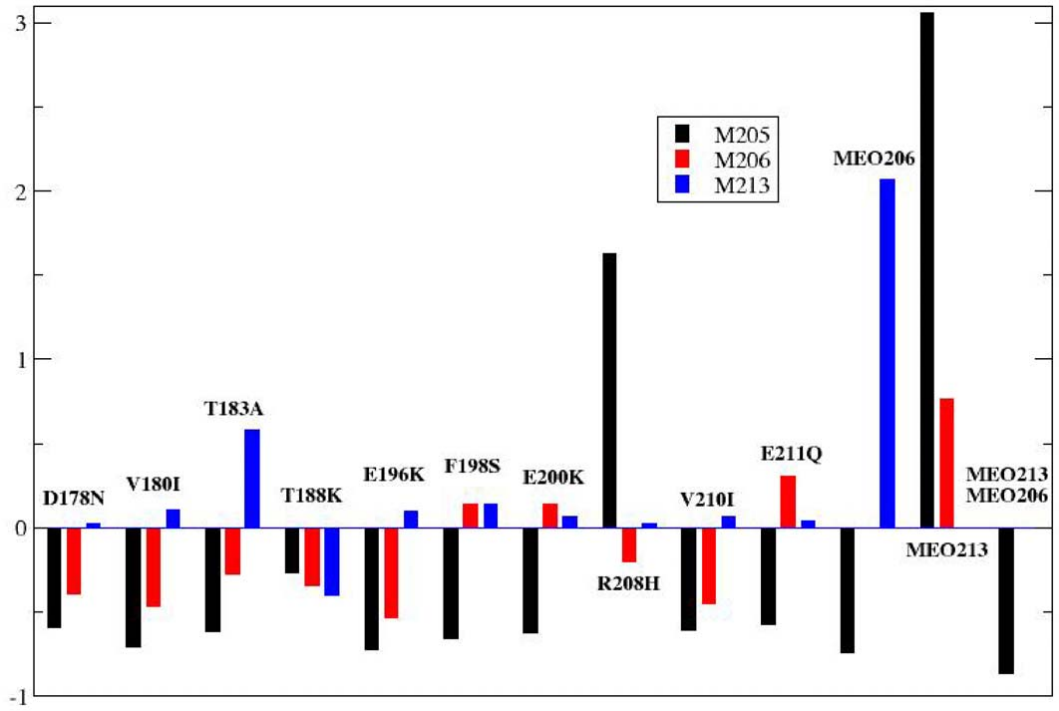
Effect of mutations and covalent modifications on the solvent exposure of α3-methionines. Displayed SASA values for M205, M206 and M213 correspond to the difference SASA_mut_ –SASA_wt_, where mut refers to mutant or covalently modified PrP chain and wt to wild type.

To gain insights into the effect of the different disease-linked mutations on the solvent exposure of α3 methionines, we analyzed the mutation-induced variation on the relative Solvent Accessible Surface Area (SASA). The SASA for residues M205, M206 and M213 was calculated by averaging along the whole simulation time for each mutant and for the wt protein. The average SASA value for the wt was subsequently subtracted from that of the mutant. A positive variation indicates an increase of the solvent exposure of M205, M206 or M213 with respect to the wt, while a negative value reports on a decrease of solvent exposure. **Figure 4** shows that except for the T188K, the presence of a mutation is correlated to an increase of the solvent exposure of M213. This increase varies in relative value from 0.1 in D178N to 1.4 in R208H. In contrast, except for F198S, E200K and E211Q, most of the mutations result in a decrease of the SASA for M206. Regarding M205, mutations other than R208H cause a reduction in the relative SASA average value. As noted, the T188K case diverges from the rest of mutations in that it is the only one inducing a reduction of the solvent exposure of all three analyzed methionines. It is worth underlining that this is an average effect. Indeed, analysis of the time dependence of the solvent accessible area for M213, M205 and M205 reveals large fluctuations, with peaks corresponding to a high exposure of the three oxidation-sensitive side chains (data not shown). However, such fluctuations are averaged by the engagement of the mutant side chain (K188) in a set of electrostatic intramolecular interactions with carboxylate groups of E196, E200 and E207, while the rest of the protein responds with increased flexibility and changes in dynamic coordination as shown above. These interactions, that are pH-dependent, then determine an average decrease of the solvent accessible surface.

Since mutations affect differently the solvent exposure of the three α3 methionines, we then asked how the exposure to solvent of a single methionine would influence the exposure of the other two. Taking into account that except for T188K, mutations increase the exposure of M213 and assuming that once exposed it becomes oxidized, to answer this question we analyzed the SASA variation of M205 and M206 using the MO213 trajectories [**Figure 4**]. Interestingly, oxidation of M213 determines the largest relative increase in the accessible surface area of M205 and M206, compared to all the disease-linked mutations. Then, these data suggest that out of the three α3 methionines M213 may be more accessible to oxidation than its neighboring M205 and M206, resulting in a kinetically faster modification of the side chain polarity for M213. This in turn leads to larger accessibility for M205 and M206 to possible oxidizing agents, allowing the oxidation of the three α3 methionine residues. Indeed, the occurrence of an increased sensitivity to oxidation in α3 methionines in mutant chains was shown using immunological tools and recombinant full length HuPrP wt and E200K chains [10]. On the other hand, given the intolerance of the α-fold for polar substitutions at M205, M206 and M213, the facilitated oxidation of α3 methionines could also explain the folding intermediate features of some PrP mutant chains [20]–[23],[31],[33],[36].

It must be noted at this point, that the relationship between MD and the experimental data considered is at most qualitative, so that these results should be considered as the basis for further experimental investigations to establish whether methionine oxidation and its effect on aggregation has a real role in the pathogenic conversion.

### Mutations remodel the surface interacting properties of the PrP α-fold

An essential aspect of the pathogenic misfolding of PrP^C^ is related to the putative remodeling of sites involved in molecular recognition events such as protein-protein and protein-ligand interactions. Despite the lack of knowledge of the partners, the pathogenic misfolding process can be conceived as an event in which the sites acting as conversion inhibitors are inactivated and those acting as effectors (as those involved in the self-assembly process) are formed. To investigate this issue we applied the recently developed MLCE method [37]. This computational approach allows the prediction of protein-protein recognition sites from the analysis of MD-based protein energetics. The method is based on the rationale that interaction sites are generally characterized by non-optimized intramolecular interaction networks, so that they may undergo conformational changes and be recognized by a binding partner with minimal energetic expense. It must be noted here that, as a consequence of its physical formulation, the MLCE method does not make a distinction between small-molecule binding sites or protein-protein interactions sites, identifying all sites where an interaction with a second molecule is possible or favourable. Interestingly, these interaction sites usually correspond to localized sub-structures of the protein that are exposed or easily accessible on the protein surface. In this context, by perturbing the intramolecular energy of interaction networks, mutations can not only affect the stability but also the recognition properties of PrP [12],[18],[19],[28]–[31],[37].

**Figure 5:**
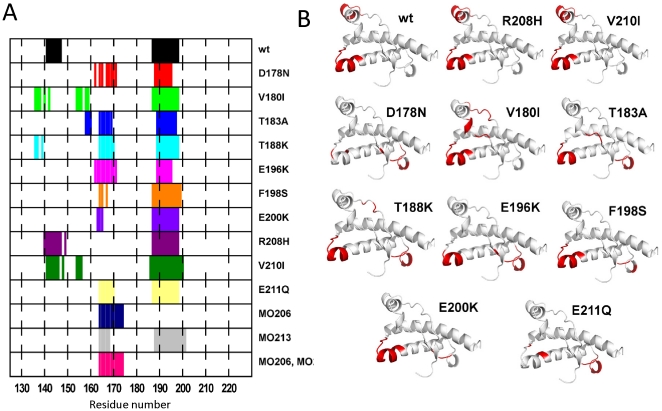
Effect of mutations on the reactive regions and sites of HuPrP(125–228). (**A**) Prediction of recognition regions by the MCLE analysis. The regions fulfilling the criteria for acting as interacting sites are depicted by colored boxes at their sequence position. The chain is indicated at the right. (**B**) 3D plot of the location of regions with interaction properties in the different HuPrP(121–228) variants.

Application of the MLCE analysis to HuPrP(125–228) wt identifies two potential interacting sites at the 141–147 and 187–198 regions located in α1 and in α2, respectively [**Figure 5**]. As shown in [38]–[41], the 141–147 region is contained in the epitopes of the anti-PrP^C^ 6H4, D18, POM1 and ICSM 18 antibodies, some of which have been shown to clear cells from prions and inhibit their formation. On the other hand, the 187–198 region overlaps partially the binding site for GN8, a small molecule that acting as chemical chaperone stabilizes the native PrP^C^ conformation and prevents its conversion to PrP^Sc^ [42],[43]. Then, the MCLE analysis of the wt native state reveals two interacting interfaces for PrP^C^ ligands acting as inhibitors of the misfolding process, either through antibody binding (residues 141–147) or through chemical chaperone interference (residues 187–198).

In the mutant chains studied here, except in R208H and V210I that retain the wt pattern, the putative 141–147 interacting region observed for the wt shifts to the region 160–173, comprising the β2-α2 loop [**Figure** **5**]. In addition, mutations also affect the length and location of the second epitope of the wt (residues 187–198). This region preserved the wt trend in V180I, T188K, E200K, and E211Q mutations, is expanded in F198S and V210I, and becomes shortened in T183A, D178N and E196K. These variations may encipher changes in the specificity and affinity of the ligands binding to it. The shift from the 141–147 to the 160–173 region indicates that mutations dictate the disappearance of the binding site for proven conversion inhibitors [38],[40],[42]. On the other hand, the identity of generated interacting site has major implications in the pathogenic conversion process. Previous work has shown that the β2-α2 loop forms part of the hypothetical “protein X” epitope which regulates the efficiency of the in vivo template-driven amplification of a prion [44]. Also, synthetic peptides overlapping this region inhibit the conversion reaction [45]. In addition, this region contains the motives ruling scrapie susceptibility in ovine and forms part of the binding site for therapeutically active cationic porphyrines [46]–[48]. The β2-α2 loop also entails a conformational complexity that encodes the efficiency of the protein conversion and importantly, behaves as a motif with self-assembly properties [9],[13],[49],[50]. Then, these evidences suggest that mutations behave as effectors for the definition of possible interactions sites. Moreover, the variation in the molecular recognition properties of the interfaces may also provide a qualitative explanation to the observed variety of oligomerization mechanisms and oligomeric structures [14].

As noted, V210I and R208H mutants diverge from the rest of the mutants studied. [**Figure 5**]. Both mutants share an increased exposure of M213 and for R208H also of M205, suggesting that under an oxidative environment the α3 methionines could exist as sulfoxides [**Figure 4**]. Strikingly, the oxidation of α3 methionines determines the same shift in the putative recognition region as observed for pathogenic mutations. In this case, the interacting properties of V210I and R208H could be approximated to that of the sulfoxidized proteins, which importantly overlaps the common pattern identified for the rest of the mutations. Then, the energetics-based prediction of PrP interaction surfaces suggests that mutations cause a common variation of the putative protein-protein recognition sites, which is featured by the loss of binding sites for conversion inhibitors and the gain of an interacting region at the amyloid-forming β2-α2 loop.

## Discussion

**Figure 6:**
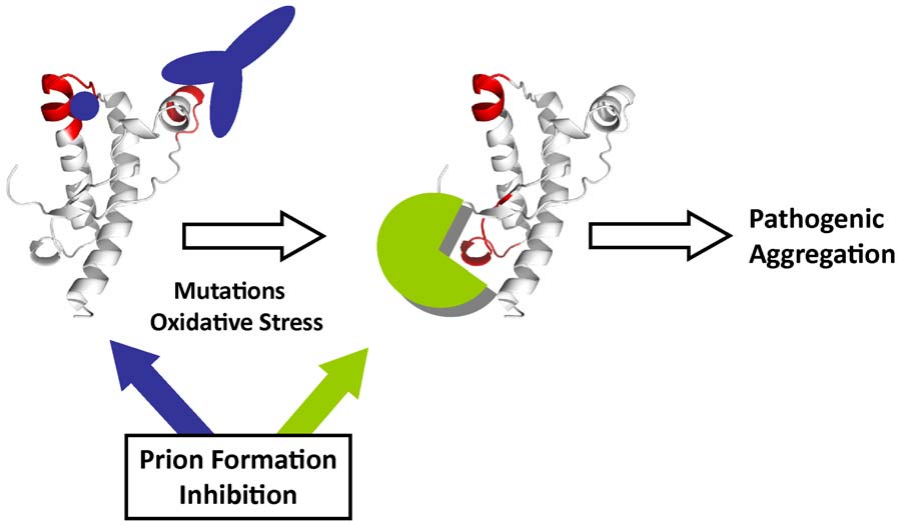
Dynamic diagnosis of PrP mutations. HuPrP(121–228) fold displays interacting sites at regions located in α1 and in α2 (depicted in red) to which antibodies and small molecules behaving as conversion inhibitors bind. Presence of a pathogenic mutation or under conditions favoring the oxidation of the α3 methionines triggers a surface reactivity remodeling featured by the emergence of reactive features at the β2-α2 loop that dictates the engagement into the pathogenic route or that can be attenuated by its use as target for interference.

Despite important advances in the last decade, the role that pathogenic mutations identified in the human PrP play in favoring the production of misfolded forms and of disease remains an open issue. Answering these mechanistic questions is essential for the design and development of prophylactic strategies for delaying the disease onset in asymptomatic carriers. Using the native state as the prevalent form and focusing on the early events, we have shown here that combining the observations from all-atom simulations of ten PrP pathogenic mutants contained in the α2-α3 region, depicts a common perturbation pathway that is supported by previous unrelated experimental findings. The obtained results show that mutations alter the flexibility and the coordination properties of the native state, with a specific impact on the dynamics of the α2-α3 helical hairpin, which in turn modify the protein interactive surface impinging on the binding sites for conversion inhibitors and activating the interacting properties of the β2-α2 loop [**Figure 6**].

While the protein is still in the native state and therefore functional, the studied mutations perturb the conformational dynamics. As a general effect and in agreement with previous reports, most mutations behave as flexibility amplifiers which favors the access to otherwise impeded states [19],[30]–[32]. Importantly, the detected perturbations transmit through the protein chain to sites distant from the mutation position. In particular, the alterations are sensed by the β2-α2 loop, the C-terminal region of α2, part of the α2-α3 loop, and the C-terminal part of α3. These effects are reminiscent of those caused by the polar perturbations of α3 methionines [32],[33]. Moreover, the long range effects of sequence variation have emerged as a general property of many proteins [51]–[53]. All the affected regions are endowed with proven importance in driving fibrillation processes [4],[8],[14],[15]. The β2-α2 loop, which links the two halves of the native fold, contains a motif that drives amyloid formation when inserted in an unrelated protein [8],[9]. The other two regions are contained in the α2-α3 subdomain, which participates in the backbone of recombinant PrP fibrils [16],[17]. Interestingly, the α2-α3 subdomain was also identified as the most relevant region for PrP oligomerization. Despite the presence of two sequences undergoing fast fibrillation, the α2-α3 hairpin behaves as a stable, independently structurally stable folded unit when isolated from the context of the whole protein [4],[14],[15]. Furthermore, chromatography and light scattering experiments showed that the α2-α3 oligomerization pattern recapitulate that of the full-length protein [14]. Moreover, except for T188K, mutations also increase the average solvent exposure of M213 compared to the wt, a factor that may facilitate the oxidation of its side chain by external agents. In turn, M213 oxidation facilitates the oxidation of M205 and M206, all immunologically proven as covalent features of aggregated human PrP chains [34]–[36]. The quantitative determination of the amounts of oxidized protein, the relevance of the in vitro experimental conditions to the in vivo situation and of this factor as a trigger for pathogenic conversion, are still open issues that need to be further investigated at the mechanistic, biochemical and structural levels. MD based results thus constitute a working hypothesis for new investigations to ascertain the role of covalent modifications in PrP pathogenic pathways.

All the changes in the native state dynamics induced by the mutations reverberate as a reorganization of reactive regions, sites entailing the recognition of small molecules, antibodies or self-association events. In the wt form the interaction hot-spots appear at the 141–147 and 187–198 regions, both proven sites for the binding of conversion inhibitors [38] [40] [42]–[43]. In contrast, in the mutants, either directly or as consequence of a facilitated putative M213 oxidation as for V210I and R208H, the interacting regions change. This change is featured by the disappearance of the 141–147 inhibitor binding site and the emergence of the 166–179 stretch (β2-α2 loop) as interacting site. The capacity of the β2-α2 loop to participate in both heterologous and homologous intermolecular interaction has been experimentally shown validating the site reactivity [9],[39],[44]. On the other hand, the inhibition of wt PrP conversion reactions by synthetic peptides overlapping the β2-α2 loop suggests that the acquisition of binding properties by this loop is a general step in the prion formation and validates its behavior as pharmacological target [45].

Our results have also important mechanistic implications for the initial steps of the pathogenic conversion process. According to Rezaei and coworkers [15], the oligomerization process of PrP would start with the disruption of the interactions of the α2-α3 helical hairpin with the rest of the domain, and the exposure of the oligomerization seeding elements. In this framework, our theoretical results show that pathogenic mutations facilitate the disruption of such interactions, acting as effectors for the detachment process. Since most mutations increase the solvent exposure of α3 methionines, possibly easing their oxidation, and polar substitutions of those side chains impair the native tertiary structure, it seems plausible to hypothesize that sulfoxidation may be simply chemically tagging the detachment and allowing its irreversibility provided the impairment of its enzymatic reduction [33],[36],[54],[55]. Indeed, full-length chains with M206S and M213S mutations oligomerize in the absence of denaturants as the isolate α2-α3 hairpin [14],[15],[33]. The α2-α3 hairpin independence is accompanied by the gain of intermolecular recognition properties at the β2-α2 loop. By acting either as a fibrillation motif or as part of the “protein X” epitope, the reactivity of the β2-α2 loop may dictate the structural transition outcome [8],[9],[44].

This dynamic and energetic model of PrP^C^ conformational perturbation corroborates these experimental observations: mutations alter the properties of the native state to different extents so that unfolding can take different pathways, giving rise to different conformations of partially unfolded states and/or populating different intermediates as found in the folding kinetics studies [23]. The observed differential perturbations of the native state dynamic properties and reactive surfaces may thus account for the accessibility to different PrP^Sc^ conformers and the possibility to form structurally different oligomers resulting from distinct kinetic pathways [56]. This would also explain the lack of correlation between the different stability of the native state and of the pathogenic conversion susceptibility in PrP mutant chains [18]. Despite its early obligatory character, the perturbation in the dynamics and consequent destabilization of native state does not provide the full molecular picture for the pathogenic conversion of PrP^C^. Polymerization, which is correlated to the stability of each of the species involved (PrP^Sc^ conformers forming pathogenic oligomers, heterogeneous oligomers, etc), plays an important role. In this context, the full characterization of the conformational preferences of the PrP^Sc^ conformers involved and the understanding of the effects of mutations on their stabilities is required. PrP^Sc^ conformers with different (relative) stabilities can in fact yield assemblies that have different quaternary organizations, solubility and toxicity. This aspect is however out of the reach of the simulations presented here. In order to apply our approach to PrP^Sc^, reliable models of the respective structures should be available. A step into this direction may be represented by the study of Chakroun *et al*. [14], whereby the structural properties of the isolate α2-α3 region, which recapitulates the amyloidogenic features of the whole protein and contains the mutations herein studied, have been elucidated by means of MD, generating a as the isolate β-sheet rich conformation consistent with experimental observations. In any case, the generation and validation of yet unavailable reliable structural models is an absolute requirement.

Overall our results show that the perturbations impinged by mutations on the native PrP^C^ native state are dynamic in nature and converge to the generation of a novel interactive patch at the region constituted by the β2-α2 loop that due to its proven interacting capacity could be used for targeted interference and provide a valid strategy to interfere with the pathogenic conversion in mutant carriers.

## Materials and Methods

### Molecular dynamics

The 3D structure of the HuPrP(125–228) (Pdb entry 1QLZ) was used as a starting point in the simulations [11]. Mutants were generated by side chain replacement using the MUTATE command of the WHATIF package [33],[57]. The oxidation variants at the different Met residues were generated as described [32]. For each system, two independent long-time scale all-atom molecular dynamics (MD) simulations in explicit water at 310K, with simulation times of at least 100 were independently run using different sets of initial velocities. The analysis was carried out on the total trajectory obtained by the combination of the two simulations for each system. The details on the force fields and on the simulations are reported in the supporting information (Supplementary Materials S1).

### Analysis of signal propagation

The analysis of the signal propagation and internal dynamic coordination as a function of the sequence changes was performed as described [32]–[33]. The communication propensity (CP) of any pair of residues is defined as the mean-square fluctuation of the inter-residue distance, defining *d_ij_* as distance between the Cα atoms of residue *i* and residue *j*, respectively:
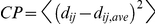



Projection of these quantities on the 3D structures of the protein allows the evaluation of differences in the intra-protein dynamical redistributions of interactions. For discriminating fast communications the threshold was set at CP = 0.25 and used a histogram representation to evaluate the signaling behavior of all amino acids in the protein.

### Prediction of protein interaction surfaces and epitope identification

The identification of putative protein interaction surfaces was carried out using the MLCE method [37]. As described in the supporting information (Supplementary Materials S1), this approach to define interaction sites stems from topological and energetic considerations. Protein substructures involved in the recognition of binding partners define continuous patches on the surface and can be characterized by the ability to visit multiple conformations, a subset of which may be optimally selected by the binding partner. In this frame of thought, the sites that constitute an interaction patch are contiguous in space and are characterized by non-optimized (minimal) energetic-couplings with other residues in the protein. The map of pair energy-couplings filtered with topological information obtainable from the contact map can thus be used to identify local couplings characterized by energetic interactions of minimal intensities. While low intensity couplings between distant residues in the structure are a trivial consequence of the distance-dependence of energy functions, local low energy couplings identify sites whose interaction-networks are not energetically optimized.

### Solvent accessible surface area (SASA) analysis

The SASA values for all the protein residues were calculated using the program ICM [Molsoft LLC, http://www.molsoft.com]. For every trajectory, one snapshot every 100 ps was extracted and SASA values were calculated using the “surface area” function of ICM package, the mean values were obtained by simple arithmetic average calculation.

## Supporting Information

Materials S1 (DOC)
