# Supplementary material for: Dynamic Diagnosis of Familial Prion Diseases Supports the β2-α2 Loop as a Universal Interference Target
Source: PLoS One. 2011 Apr 28;6(4):e19093. doi: 10.1371/journal.pone.0019093 (PMC3084259; doi:10.1371/journal.pone.0019093)
Supplement: Materials S1 — (DOC) [file pone.0019093.s001.doc]

**Supplementary Materials and Methods**

**MD Simulation set up**. The convergence of each simulation, within the limitations of the simulation timescales, was checked by calculating conformational and energetic properties in time windows of increasing lengths [**1**]. All simulations and the analysis of the trajectories were performed by using the GROMACS software package [**3**], using the GROMOS96 force field [**4**] and the SPC water model [**5**]. The same calculation set up was used for each simulation. To mimic proper solution conditions, the ε-amino groups were considered protonated, while the carboxyl groups were considered to bear a negative charge. The systems were solvated in an octahedral-shaped box large enough to contain 0.9 nm of solvent around each aggregate. Each system was subsequently energy minimized with a steepest descent method for 2000 steps. The calculation of electrostatic forces utilized the PME implementation of the Ewald summation method. The constraining of all bond lengths was performed with the LINCS algorithm [**6**]. A dielectric permittivity, ε = 1, and a time step of 2 fs were used. All atoms were given an initial velocity obtained from a Maxwellian distribution at the desired initial temperature of 310K. Different initial random seeds were used to start different simulations at a certain temperature. The density of the system was adjusted performing the first equilibration runs at NPT condition by weak coupling to a bath of constant pressure (P_0_ = 1 bar, coupling time τ*_p_ =* 0.5 ps) [**7**]. In all simulations the temperature was maintained close to the intended values by weak coupling to an external temperature bath with a coupling constant of 0.1 ps [**7**]. The proteins and the rest of the system were coupled separately to the temperature bath.

**Protein-Protein Interaction Patches and Epitope Identification.** In the approach we use herein, the analysis of energetics is based on our Energy Decomposition method that allows detecting residue-couplings important in the stabilization of a fold. The method provides a simplified view of residue-residue pair interactions, extracting the major contributions to energetic stability of the native structure from the results of all-atom MD simulations. For a protein of *N* residues, the *N*x*N* matrix (*M_ij_*) of average non-bonded interactions between pairs of residues can be built by averaging over the structures visited during an MD trajectory [**8-12**]. The rather noisy energy matrix is then simplified through eigenvalue decomposition. Analysis of the *N* components of the eigenvector associated with the lowest eigenvalue was shown to identify residues behaving as strong interaction centres, characterized by components with intensity higher than the threshold value corresponding to a “flat” normalized vector whose residues would all provide the same contribution. The method was validated against experimental data and a relationship was found between the topological and energetic properties of a protein and its stability [**8-12**]

The matrix *M_ij_* is thus diagonalized and re-expressed in terms of eigenvalues and eigenvectors, in the form:

$M_{ij}=\sum_{k=1}^{N} \lambda_{k}w_{i}^{k}w_{j}^{k}$ (1)

where *N* is the number of protein aminoacids, $\lambda_{k}$is the *k*-th eigenvalue with *k* ranging from 1 to *N*, and $w_{i}^{k}$, $w_{j}^{k}$ $w_{i}^{k}$are the *i-*th and *j-*th components of the associated normalized eigenvector. Eigenvalues are labelled following an increasing order, so that $\lambda_{1}$is the most negative. In the following we refer to the first eigenvector as the eigenvector corresponding to eigenvalue $\lambda_{1}$. The total non-bonded energy *E_nb_* is defined as:

$E_{nb}=\sum_{i,j=1}^{N} M_{ij}=\sum_{i,j=1}^{N} \sum_{k=1}^{N} \lambda_{k}w_{i}^{k}w^{k}_{j}$ (2)

We showed [**8,12**] that each *M_ij_* can be effectively approximated by:

$M_{ij}\approx\tilde{M}_{ij}=\lambda_{1}w_{i}^{1}w_{j}^{1}$ (3)

such that the total non bonded energy becomes:

$E_{nb}\approx E^{app}_{nb}=\sum_{i,j=1}^{N} \tilde{M}_{ij}=\sum_{i,j=1}^{N} \lambda_{1}w_{i}^{1}w^{1}_{j}$ (4)

The principal eigenvector (defined the Sequence Eigenvector, SE) constitutes a simple vectorial representation of the sequence: it reports on the contribution of each residue in the stabilization of the fold, which ultimately depends on the chemical properties of the residue itself. From this we can recover an approximation to the global stabilization energy, *E_nb_^app^*, that was shown to correlate with the relative different stabilities of mutants of several proteins, proving the sufficient energetic descriptor to discriminate among them [**12**]. This method provides information on the mean coupling energy between two residues in the native state, revealing the network of most interacting residues through the structure.

The contact map of the representative structure from MD recapitulates which residue pairs are in contact in the conformation. If the distance between any two C_β_ atoms is below a cut-off value, the corresponding matrix entry is set to 1, otherwise it is set to 0. The distance cut-off is set to 6.5Å. For the sake of homogeneity with the energy matrix, also contacts between nearest neighbours *i, i+1* are included. Therefore:

$C_{ij}=\left\{\begin{matrix} 1 & r_{ij}\leq6.5 \\ 0 & ri_{j}>6.5 \end{matrix}\right.$ (5)

To calculate the contact matrices we consider the representative structure of the main cluster obtained with the GROMOS method from the MD trajectory of each antigen (cut-off value of 2Å) [**13**]. Energy decomposition was carried out both by averaging on structures saved every ns during the simulations and on the representative protein conformation of the most populated structural cluster obtained from the trajectory. The resulting structures were minimized, and solvation effects were taken into account using the MM-PBSA method, with the non-bonded energy term for residues *i* and *j* resulting *E_ij_^nb^ = E_elect,ij_ + E_vdW,ij_ + G_solv,ij_* [**14**]. The results of averaging over the trajectory and of considering the most populated cluster are basically identical.

**Interaction Surface (Epitope) Identification***.* The simplified interaction matrix defined by $\lambda_{1}w_{i}^{1}w^{1}_{j}$ is multiplied (through the Hadamard product) by the residue-contact matrix. This procedure allows to filter the information contained in the simplified energy matrix $\lambda_{1}w_{i}^{1}w^{1}_{j}$ in terms of residues that are close in space, highlighting pairs within the contact cut-off that are also coupled through non-bonded interactions. This provides a compact way to highlight which local pair-contacts in the 3D organization of the protein are coupled through energetic interactions. The resulting matrix can be viewed as the *Matrix* *of Local Coupling Energies (MLCE)*. The contact-filtered coupling interactions are ranked in increasing order according to their respective intensities (from weaker to stronger). Starting from the minimum, the set of putative interaction sites is defined by including increasing residue-residue coupling values until the number of couplings corresponding to the lowest 15% of all contact-filtered pairs was reached. This corresponds in our approximation to the set of local interactions with minimal intensities that may identify epitopes or protein interaction sites.

**References**

1. Colombo G, Meli M, Morra G, Gabizon R, et al. (2009) [Methionine sulfoxides on prion protein Helix-3 switch on the alpha-fold destabilization required for conversion.](http://www.ncbi.nlm.nih.gov/pubmed/19172188) *PLoS One* 4, e4296
2. Vriend G (1990) What if: a molecular modeling and drug design program. *J Mol Graph* 8, 52–56
3. Lindahl E, Hess B, van der Spoel D (2001) Gromacs 3.0: A package for molecular simulation and trajectory analysis. *J Mol Mod* 7, 306–317
4. van Gusteren WF, Daura X, Mark AE (2006) Gromos force field. *Encyclopedia of Computational Chemistry* 2, 1211–1216
5. Berendsen HJC, Grigera JR, Straatsma PR (1987) The missing term in effective pair potentials. *J Phys Chem* 91, 6269–6271
6. Hess B, Bekker H, Fraaije JGEM, Berendsen HJC (1997) A linear constraint solver for molecular simulations. J Comp Chem 18: 1463–1472
7. Berendsen HJC, Postma JPM, van Gusteren WF, Di Nola A, Haak JR (1984) Molecular dynamics with coupling to an external bath. *J Chem Phys* 81, 3684–3690
8. Tiana G, Simona F, De Mori GM, Broglia RA, Colombo G. (2004) Understanding the determinants of stability and holding of small globular proteins from their energetics. *Protein Sci.* 13, 113-124
9. Ragona L., Colombo G., Catalano M., Molinari H. (2005)Determinants of protein stability and folding: comparative analysis of *β*-lactoglobulins and liver basic fatty acid binding protein, *Proteins: Struct. Funct. and Bioinf.* **61,** 366–376
10. [Colacino S, Tiana G, Broglia RA, Colombo G.](http://www.ncbi.nlm.nih.gov/pubmed/16432880?ordinalpos=2&itool=EntrezSystem2.PEntrez.Pubmed.Pubmed_ResultsPanel.Pubmed_DefaultReportPanel.Pubmed_RVDocSum) (2006) The determinants of stability in the human prion protein: insights into folding and misfolding from the analysis of the change in the stabilization energy distribution in different conditions. *Proteins* 62, 698-707.
11. Morra G, Colombo G. (2008) Relationship between energy distribution and fold stability: insights from molecular dynamics simulations of native and mutant proteins. *Proteins* 72, 660-672.
12. Colombo G, Morra G, Meli M, Verkhivker G.(2008) [Understanding ligand-based modulation of the Hsp90 molecular chaperone dynamics at atomic resolution.](http://www.ncbi.nlm.nih.gov/pubmed/18511558) *Proc Natl Acad Sci U S A.* 105, 7976-7981.
13. Daura X., Gademann K., Jaun B., Seebach D., van Gusteren WF, et al. (1999) Peptide folding: when simulation meets experiment, *Angew. Chemie Intl. Ed.* **38**, 236–240.
14. Wang W. Lim WA, Jakalian A., Wang J., Wang J., et al. (2001) An analysis of the interactions between the Sem-5 SH3 domain and its ligands using molecular dynamics, free energy calculations, and sequence analysis, *J. Am. Chem. Soc.* **123**, 3986-3994
